# LGR5: An emerging therapeutic target for cancer metastasis and chemotherapy resistance

**DOI:** 10.1007/s10555-024-10239-x

**Published:** 2025-01-17

**Authors:** Wanqi Wang, Noor A. Lokman, Simon C. Barry, Martin K. Oehler, Carmela Ricciardelli

**Affiliations:** 1 Discipline of Obstetrics and Gynaecology, Adelaide Medical School, Robinson Research Institute, The University of Adelaide, Adelaide, 5005 Australia; 2 Molecular Immunology, Robinson Research Institute, University of Adelaide, Adelaide, 5005 Australia; 3 Department of Gynaecological Oncology, Royal Adelaide Hospital, Adelaide, 5000 Australia

**Keywords:** LGR5, WNT/β-catenin, Metastasis, Chemoresistant

## Abstract

Cancer stem cells play an important role in tumor progression and chemotherapy resistance. Leucine-rich G repeat-containing protein-coupled receptor 5 (LGR5) has been identified as a cancer stem cell marker in several cancer types. LGR5 is involved in cancer development and progression via several pathways including WNT/β-catenin signaling pathway. LGR5 plays a role in tumor progression by promoting cancer cell migration, invasion, metastasis, and angiogenesis in many cancers including colorectal, brain, gastric, and ovarian cancer. This review summarises the current knowledge on the expression and functional role of LGR5 in cancers, the molecular mechanisms regulated by LGR5, and the relationship between LGR5 and chemotherapy resistance. The review also includes highlights potential strategies to inhibit LGR5 expression and function. The majority of functional studies have shown that LGR5 plays an important role in promoting cancer progression, metastasis and chemotherapy resistance however, in some contexts LGR5 can also activate tumor-suppressive pathways and LGR5 negative cells can also promote cancer progression. The review highlights that targeting LGR5 is a promising anti-cancer treatment but the functional effect of LGR5 on tumor cells is complex may be dependent on cancer type, tumor microenvironment and cross-talk with other molecules in the LGR5 signaling pathway.

## Introduction

Cancer continues to be one of the most difficult diseases to combat globally, despite extensive research and recent advancements in systemic treatments. Generally, the majority of cancer-related deaths are due to tumor recurrence and metastasis [1]. Thus, it is crucial to have deeper understanding of the cellular and molecular mechanisms that drive cancer recurrence and spread.

Cancer stem cells (CSCs) are tumor-initiating or propagating cells that play a role in tumor progression, chemotherapy resistance, and recurrence [2, 3]. CSCs have unique ability to self-renew and differentiate, forming diverse cell populations within tumors. CSCs are central to cancer progression due to their capacity to maintain tumor growth and contribute to its heterogeneity [4]. It is widely believed that CSCs are involved in cellular reprogramming during oncogenic transformation, and drugs targeting CSCs will inhibit cancer progression and tumor self-renewal [2]. CSCs are pro-tumorigenic and have been identified in several malignancies [2, 5]. Chemotherapy resistance is a major challenge in cancer treatment, and CSCs are more resilient to chemotherapeutic agents due to several mechanisms. These include the overexpression of ATP-binding cassette (ABC) transporters, which pump drugs out of the cells, enhanced DNA repair capabilities, activation of anti-apoptotic pathways, and the quiescent nature of CSCs [6, 7]. Mesenchymal stem cells (MSCs) can regulate CSCs and contribute to drug resistance by secreting extracellular vesicles, cytokines, and growth factors, promoting stemness and enhancing survival under therapeutic stress [7]. MSCs shape the tumor microenvironment (TME) by fostering immunosuppression, angiogenesis, and epithelial-mesenchymal transition (EMT), further promoting therapy resistance [7].

Following initial treatment, the persistence of CSCs is a key factor in tumor recurrence. Even after successful chemotherapy, surviving CSCs can re-enter the cell cycle, leading to the regeneration of the tumor, often with more aggressive characteristics [8]. The ability of CSCs to repopulate the tumor underscores the importance of developing therapies that specifically target these cells to prevent relapse [9]. Understanding the role of CSCs in cancer progression, therapy resistance, and recurrence is crucial for improving cancer treatment outcomes and reducing the likelihood of recurrence.

Leucine-rich G repeat-containing protein-coupled receptor 5 (LGR5), has been identified as a stem cell or progenitor cell marker in normal tissues and many cancers, [10–13]. This review discusses the current literature on the expression and functional roles of LGR5 in cancers and chemotherapy resistance and highlights strategies to inhibit LGR5 in cancer.

## LGR5 structure and function

LGR5, also known as G-protein coupled receptor 49 or 67 (GPR49 or GPR67), is a G-protein coupled receptor (GPCR), one of the largest membrane protein families encoding more than 800 human genes. LGR5 belongs to class A (rhodopsin-like) subgroup of GPCRs and is a part of LGR (LGR4-6) subfamily [8]. LGR receptors including LGR5 are known for their involvement in the regulation of stem cells and tissue homeostasis [8]. In general, GPCRs are critical signal transducers that control vital physiological functions involving smooth muscle contraction, blood pressure regulation, neurotransmission, hormone, and immune responses [9]. Up to 50–60% of the current therapeutic agents either directly or indirectly target GPCRs and over 30% of all Food and Drug Administration approved drugs target GPCRs [14].

### LGR5 structure

**Fig. 1:**
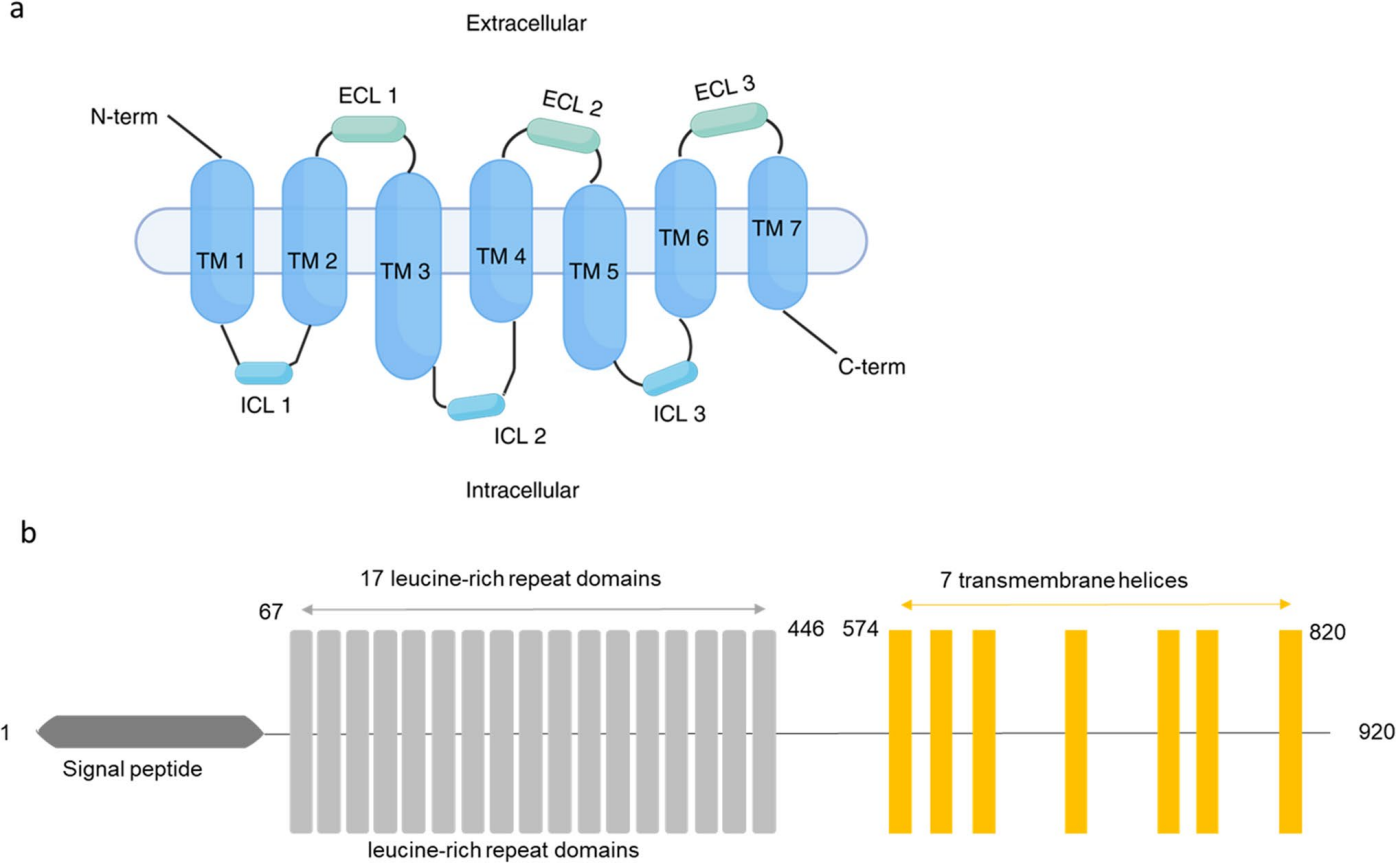
LGR5 Structure. (**a**) General architecture of G-protein coupled receptors (GPCRs) consists of an N-terminal domain attached to the 3 intracellular loops (ICL1-ICL3, blue) and three extracellular loops (ECL1-ECL3, green) followed by the C-terminal domain. (**b**) LGR5 consists of a signal peptide (dark grey), a large N-terminal domain with 17 leucine-rich repeats (grey) and 7 transmembrane helices (yellow). Modified from *Kumar K.K. 2014* [16]. Created in *BioRender*. Wang, J. (2024) https://BioRender.com/e54f457

GPCRs include an N-terminal extracellular domain, following with three intracellular loops (ICL1-ICL3), a transmembrane region involving 7 hydrophobic α-helical transmembrane domains responsible for signal transduction, and three extracellular loops (ECL1-ECL3) followed by the C-terminal domain (Fig. 1a). Unlike other GPCRs, LGR5 is a member of the glycoprotein hormone receptor subfamily which are known for their involvement in the regulation of stem cells and tissue homeostasis. The human LGR5 gene consists of 144,810 bases and is located on chromosome 12, specifically at position 12q22-q23 [15]. The LGR5 structure is shown on Fig. 1b**.** LGR5 protein (100kDa) is conserved across humans, rats, and mice, with all homologs consisting of 907 amino acids. During protein synthesis, the signal peptide (amino acids 1–21) is cleaved, leaving the mature peptide (amino acids 22–907) [15]. This mature form integrates its transmembrane domains into the transposon membrane before being processed and trafficked to the plasma membrane. The 17 leucine-rich repeat (LRR) domains starting from no.67 amino acid are the hallmark of LGR5 that distinguishes LGR5 from other GPCR family members (Fig. 1b) [16]. LGR5 forms a curved solenoid structure with the 17 LRR domains structure and does not interact with small-molecule ligands, hormones, or neurotransmitters like typical GPCRs [16].

### The role of LGR5 in the WNT/β-catenin signaling pathway

The WNT signaling pathway is crucial for embryonic development tissue regeneration, cancer development, and progression [17]. WNT proteins activate two major intracellular pathways, canonical and non-canonical signaling pathways [17]. The canonical WNT/β-catenin pathway can regulate the stemness, proliferation, and differentiation of several adult stem cells niches, such as stem cells at the intestinal crypt, hematopoietic tissues and mammary glands [17]. The canonical WNT/β-catenin signaling pathway is also involved in the progression of several human cancers [10, 18].

**Fig. 2:**
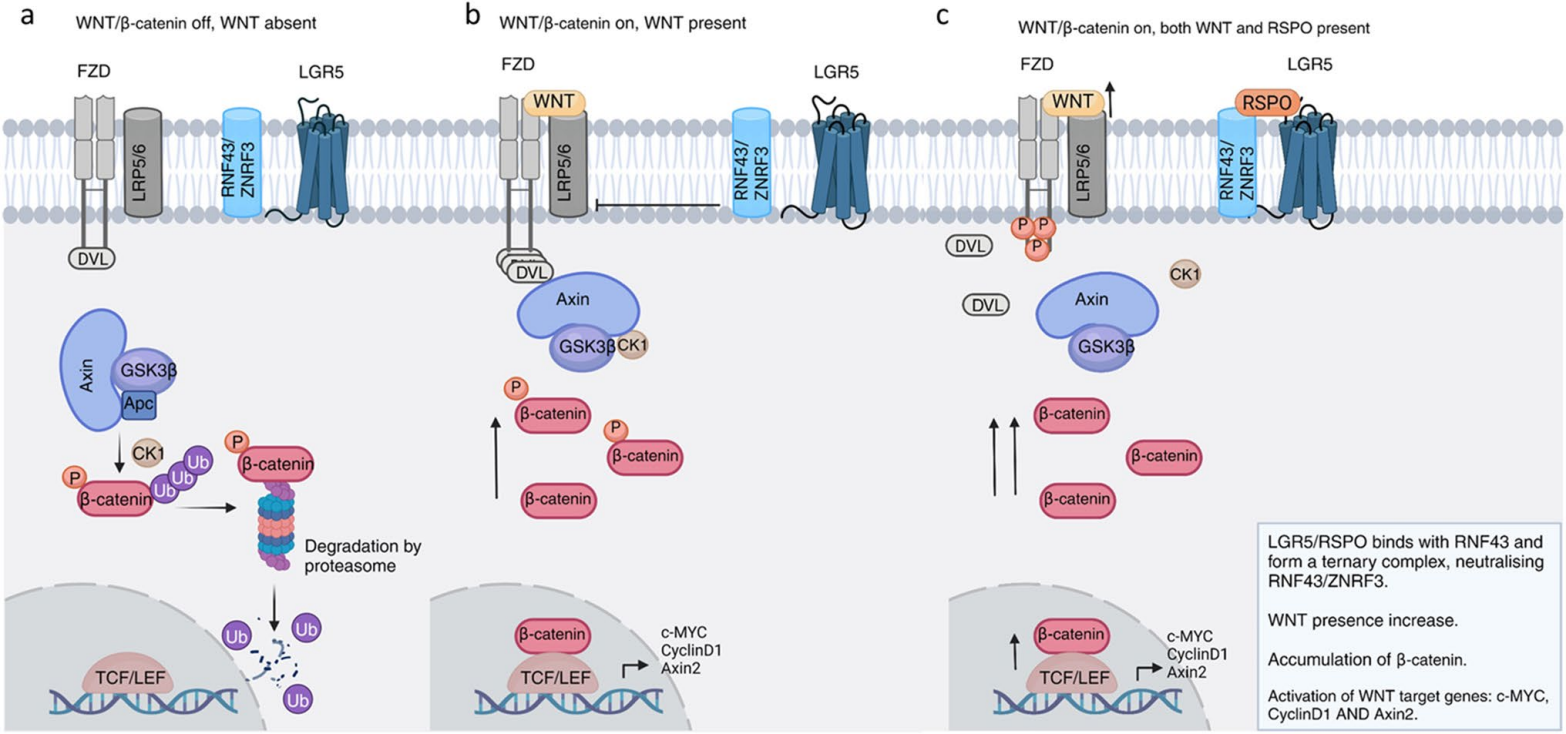
The role of LGR5 in the WNT/β-catenin pathway. (**a**) In absence of WNT, the canonical WNT/β-catenin signaling pathway is off. Central destruction complex including Axin, Glycogen Synthase Kinase 3 Beta (GSK3 β), Adenomatous Polyposis Coli (APC) and Casein kinase 1 (CK1) induce the degradation of β-catenin. (**b**) In the presence of WNT, WNT binds to receptors Frizzled (FZD) and Low-Density Lipoprotein Receptor Related Protein 5 and 6 (LRP5/6), inducing dissociation of β-catenin from central destruction complex, which leads to accumulation of β-catenin. β-catenin binds to transcription factors TFC/LEF and activation of proto-oncogenic WNT target genes (c-MYC, CyclinD1 and Axin2). RING finger protein 43 (RNF43) and its homolog Zinc/Ring finger protein 3 (ZNRF3) negatively regulate WNT pathway by internalizing and degrading FZD and LRP5/6. (**c**) In the presence of R-spondin (RSPO), LGR5 binds to RSPO, RNF43/ZNRF3 is neutralized, and intracellular β-catenin is accumulated and increased, leading to enhanced activation of the WNT signaling pathway. Created in BioRender. Wang, J. (2024) https://BioRender.com/a56w665

LGR5 has been identified as a stem cell-specific receptor to promote the canonical WNT/β-catenin signaling pathway, in cooperation with WNT protein (Fig. 2). In the absence of WNT, WNT/β-catenin signaling is off. The central destruction complex (Axin, GSKβ, Apc, CK1) induces β-catenin degradation, restraining transcription of WNT target genes (c-MYC, CyclinD1, Axin2) (Fig. 2a). When WNT is present in the absence of LGR5 ligand, WNT binds to FZD and LRP5/6, WNT/β-catenin signaling is on (Fig 2b). This causes phosphorylation of the LRP5/6 receptor and degradation of intracellular GSK3β (Glycogen synthase kinase-3 beta) which migrates to the nucleus and forms a complex – TCF/LEF (T-cell factor/lymphoid enhancer-binding factor). Dissociation of β-catenin is induced from the central destruction complex, leading to β-catenin accumulation, nuclear translocation, and transcription of WNT target genes (Fig. 2b). In this situation, RNF43/ZNRF3 negatively regulates WNT/β-catenin pathway by degrading membrane receptors FZD and LRP5/6 (Fig. 2b) [10].

In the presence of R-spondin (RSPO, the LGR5 N-terminal domain binds with RSPO and interacts with the WNT ligands, sustaining WNT/β-catenin signaling by neutralizing RNF43/ZNRF3 ligases (Fig. 2c) [16]. WNT can no longer be removed from cell membrane, which continuously increases the accumulation of β-catenin and the activation of WNT ligand-mediated pathway (Fig. 2c). The C-terminal domain of LGR5 also plays a role in stabilizing β-catenin by coupling with G-proteins and facilitating the transmission of signals from the extracellular environment to intracellular effectors [16]. The proper functioning of LGR5 C-terminal domain ensures the receptor's ability to participate in multiple rounds of signaling, maintaining the robustness of WNT/β-catenin signaling pathway [16].

### LGR5 as a stem cell marker in differentiation and wound repair

LGR5 is a transcriptional WNT target gene expressed in the crypt base of small and large intestines and was identified as a stem cell marker for cell differentiation in the small intestine and colon [19]. LGR5 also marks epithelial stem cells in the stomach [20], kidney [10, 21], hair follicle [10], mammary gland [10, 22], endometrium [23], and ovary [10, 24], suggesting that LGR5 is a potential universal adult epithelial stem cell marker [23, 25]. Cumulating evidence from many studies indicates that LGR5 is important for normal embryonic development [24, 26–31].

It is well-known that intestinal epithelium can continually regenerate [19]. At the base of the intestinal crypts, a small population of stem cells drives the epithelium generation [32]. *In vivo*, lineage tracing experiments were conducted to evaluate LGR5 expression in intestinal crypts [33]. LGR5 was expressed in the crypt base columnar (CBC) cells between the Paneth and base of the crypts [33]. CBC cells are in the “stem cell zone” which contains a population of wedged-shape immature cells. One intestinal study introduced lacZ into LGR5 + CBC cells [19]. LacZ + cells were initially present at the crypt base and then extended from the crypt base toward the tips of the interstitial villi [19]. Multicolor lineage tracing experiments have shown that LGR5 + stem cells divide symmetrically [34]. LGR5 + stem cells rarely generate daughter cells which adopt divergent fates in the short term [35]. However, multicolor stem cells are converted into single-color cells in the long term, gradually becoming clonal [35]. In the stomach, LGR5 + cells also contributed to long-term gastric epithelium re-growth, accompanied by overexpression of other WNT-regulated genes, such as c-Myc and CD44 [36].

Stem cells also play an essential role in wound repair due to their ability to self-renew and differentiate into various cell types required for tissue regeneration [37]. Particularly those cells marked by LGR5, have been identified in the ovarian epithelium and are thought to contribute to the rapid re-epithelialization of the ovarian surface post-ovulation and ovarian would healing [31]. LGR5 was initially found to control specific embryonic tissue differentiation and growth [32]. Ng. and colleagues identified LGR5 + cells within the ovary and tubal epithelia that possessed stem cell properties [24]. Their research showed that LGR5 + cells contributed to the maintenance and regeneration of the epithelial lining in both the ovary and the fallopian tube [24]. Further research suggested that LGR5 + stem/progenitor cells are responsible for ovarian epithelium regeneration and may be involved in ovarian cancer initiation [26].

In conclusion, LGR5 is a crucial stem cell marker involved in the maintenance and renewal of stem cells in various tissues, such as the intestines, stomach, hair follicles and the ovary. It is a target of the WNT/β-catenin signaling pathway, which is essential for regulating stem cell proliferation and tissue homeostasis. LGR5 + cells are responsible for self-renewal and differentiation into specialized cells, making them key players in both normal tissue regeneration and cancer, where they contribute to tumor growth and resistance to therapy.

## LGR5 expression in cancer

LGR5 expression is increased in many cancers compared with normal tissues [38–48]. LGR5 has been widely studied in colorectal cancer (CRC) and the majority of studies have shown that high LGR5 protein expression is related to poor survival outcome [11, 49–54]. A meta-analysis including 9 studies that assessed LGR5 protein expression by immunohistochemistry (IHC) and 3 studies that assessed *LGR5* mRNA expression found that high LGR5 levels were associated with reduced overall survival (OS) [55]. High LGR5 protein expression has also been associated with poor outcome and recurrence in cancers of the brain [56], lung [13], esophagus [39], breast [57], colon [11, 52–54], cervix [58] and ovary [47, 48]. Together these studies support the hypothesis that high LGR5 expression is associated with cancer progression and metastasis.

However, high LGR5 protein expression was also found to be a significant independent predictor of increased OS in CRC [59]. Jang *et al.* investigated *LGR5* expression using RNA *in situ* hybridization in 788 CRC tissues and found that high *LGR5* expression was associated with a better clinical outcome [60]. Two recent ovarian cancer studies also reported that high LGR5 mRNA expression was significantly associated with increased progression-free survival (PFS) in high grade serous ovarian carcinoma [61, 62]. The contradictory findings may be due to different methods and LGR5 antibodies used to assess LGR5. Interestingly, Kim et. al. (2022) also used RNA *in situ* hybridization to measure LGR5 mRNA expression in an ovarian cancer study [61]. In general, RNA *in situ* is a sensitive and specific method for detecting LGR5 expression in human formalin-fixed paraffin-embedded tissues. However, caution is necessary when interpreting results, especially in organs where LGR5 expression is extremely weak or transient, to avoid false negative findings. A recent study found that only mRNA levels of splice variant LGR5Δ5 (LGR5 lacking exon 5), but not full-length LGR5 was significantly associated with poor prognosis in oral squamous cell carcinoma [63]. Disparate finding between mRNA and protein may also be due to post-translational modifications of LGR5, including phosphorylation, glycosylation, and ubiquitination [64]. Phosphorylation regulates signal transduction pathways, while ubiquitination targets LGR5 for proteasomal degradation, affecting its stability, localization, and function [64, 65]. MicroRNAs and other non-coding RNAs potentially can bind to *LGR5* mRNA and suppress its translation into protein without degrading the mRNA [66]. Under some circumstances, local TME can affect LGR5 protein expression but not mRNA expression. Inflammation and hypoxia in tumor tissue microenvironment can also potentially inhibit LGR5 protein expression through post-translational mechanisms [67, 68]. A study has found that glucose deprivation altered the glycosylation status of LGR5, resulting in reduced protein stability and cell surface expression [69]. Patient cohort differences may also impact on whether LGR5 is associated with good or poor prognosis. A recent investigation found that high LGR5 protein levels in estrogen-negative (ER-) breast cancer were associated with reduced recurrence-free survival and OS, but interestingly, high LGR5 levels in ER + breast cancers were associated with better outcomes [70]. These findings suggest that LGR5 expression may modulate the Wnt/β-catenin pathway to varying extents based on hormone receptor status.

Overall, these studies highlight that there are some controversies on the prognostic significance of LGR5 in cancers. It is not clear whether the disparate findings between protein and mRNA assessments may be due to patient heterogeneity, specificity of antibodies used for LGR5 IHC, post-transcription regulation, expression of different splice variants of LGR5, or the stability of LGR5 mRNA in formalin-fixed paraffin-embedded tissues. Going forward it will be important to develop standardized methods for evaluating LGR5 protein levels and mRNA expression in formalin-fixed tissues. Further studies need to also investigate how post-transcription regulation and TME influence LGR5 mRNA and protein stability.

## Functional roles of LGR5 in cancer

LGR5 has been shown to play a role in promoting cancer cell proliferation, tumorigenesis, clonogenicity, adhesion, migration, invasion, metastasis and angiogenesis, and inhibiting apoptosis. Table 1 summarises the studies investigating these functional roles of LGR5 in different cancers.

**Table 1:** Summary of the functional roles of LGR5 in cancers

Functional roles	Tumor Type	Cell lines/Animal species	Ref
Proliferation and Apoptosis			
Transfection with LGR5-specific siRNA decreased proliferation capacity of primary astrocytes	Brain	Adult male Sprague–Dawley rats (*n* = 72): sham operation (*n* = 12) and contusion injury (*n* = 60)	[71]
LGR5 silencing in U87 cells suppressed cell proliferation and cell cycle and reduced tumor-sphere formation *in vitro* LGR5 depletion significantly inhibited tumor orthotropic xenograft growth *in vivo*	Brain	U87 cell line BALB/C nude mice	[72]
Silencing of *LGR5* by lentiviral shRNA-mediated knockdown induced apoptosis in brain CSCs	Brain	Brain CSC lines NCH421k and NCH441 cells	[73]
*LGR5* mRNA and protein activate the WNT/β-catenin signaling pathway and enhance cell proliferation by activating PKA	Breast	Breast cancer cell line: MCF-7	[74]
*LGR5* siRNA inhibited cell proliferation with induced apoptosis via suppressing the WNT/β-catenin signaling pathway	Breast	Breast cancer cell lines: MCF-7, MDA-MB-231, BT-549, ZR-75–1	[57]
LGR5-KD reduced cancer clonogenicity and tumorigenesis	Breast	Breast cancer cell lines: MDA-ctrl and MDA-LGR5KD	[70]
LGR5 does not promote Ewing sarcoma (ES) cell proliferation *in vitro*	Ewing sarcoma	Bone cancer cell lines: A673 and CHLA25	[43]
Elevated LGR5 expression increased tumor sphere-forming efficiency *in vitro*. LGR5 + cells were highly tumorigenic *in vivo*	Cervical	Human cervical carcinoma cell lines HeLa and SiHa Female NOD/SCID mice	[58]
LGR5 was positively correlated with cell proliferation *in vitro* and tumor formation *in vivo* by enhancing the cell cycle and WNT/β-catenin pathway	Cervical	Human cervical cancer cell lines: SiHa and HeLa cells Female BALB/c-nude mice	[41]
LGR5-KD suppressed cell proliferation and colony formation ability and enhanced apoptosis. Overexpression of LGR5 increased cell proliferation	Colorectal	Colon cancer cell lines: LOVO, HT29, HCT116	[40]
LGR5 silencing reduced proliferation, migration and colony formation, and downregulated NOTCH signaling *in vitro* and tumorigenicity *in vivo*	Colorectal	CRC cell lines: HT-29, SW480,	[75]
LGR5 expression correlated with proliferation via the WNT/β-catenin pathway in CRC tissues	Colorectal	CRC tissues (*n* = 53)	[76]
Ablation of LGR5 enhanced cell growth, tumorigenesis and clonogenicity	Colorectal	Colon cancer cell line: LIM1899	[77]
LGR5-KD decreased *APC* (adenomatous polyposis coli) expression and WNT/β-catenin pathway and inhibited cell proliferation in HT29 cells	Colorectal	Colon cancer cell line: HT29	[78]
Depleting *LGR5* inhibited proliferation via suppressing WNT signaling in CRC cells Depleting *LGR5* induced apoptosis, suppressed β-catenin nuclear translocation and blocked the activity of Wnt/β-catenin signaling	Colorectal	Colon cancer cell lines: LOVO, HT-29, and SW48	[79]
*LGR5* silencing inhibited cell proliferation, secondary tumor sphere formation and induced cell apoptosis, and G0/G1 phase *in vitro* LGR5-KD suppressed tumorigenicity of HT29 spheroid cells *in vivo*	Colorectal	Colon cancer cell line: HT-29 BALB/C-nu mice	[80]
High LGR5 protein and mRNA expression positively correlated with cell proliferation *in vitro* and *in vivo*	Colorectal	Colon cancer patient tissues (*n* = 366). C57/BL6 mice	[81]
*LGR5* KD significantly reduced proliferation and colony formation	Colorectal	Colon cancer cell line: CT-26	[82]
LGR5 was associated with the maintenance of colon cancer. RSPO2/LGR5 promotes formation and proliferation of spheroid colon cancer cells	Colorectal	Colon cancer cell line: HCT116	[83]
RSPO1/LGR5 directly activated TGFβ signaling in colon cancer, inhibiting TGFβ-mediated growth and stress-induced apoptosis. Decreased LGR5 inhibited clonogenicity and cell survival of colon cancer cells *in vitro*	Colorectal	Colon cancer cell lines: HCT116, RKO, FET, CBS, HCT116b, TENN	[84]
CASC 15 overexpression promoted colon cancer cell proliferation by activating the WNT/β-catenin pathway by upregulating LGR5 expression through microRNA-4310	Colorectal	Human colon cancer cell lines: HT29, HCT116, SW480, SW620	[85]
LGR5 was positively correlated with proliferation marker, Ki-67	Colorectal	CRC tissues (*n* = 192)	[86]
Sustained LGR5 + cell depletion is required to prevent colon tumor progression. Discontinued LGR5 + ^depletion^ led to re-initiation of tumor growth	Colorectal	Apc^min/+^, Kras^LSL−G12D/+^, Vil1^Cre^ (AKV) were crossed with LGR5^DTR/eGFP^ mice	[45]
Reduced LGR5 expression was associated with inhibited colony formation in HT29 spheroids	Colorectal	Colon cancer cell line: HT29	[87]
miR-138-5p regulates CRC progression and promotes apoptosis via the SP1/LGR5 axis	Colorectal	Colon cancer cell lines: LOVO, HCT116, SW620, COLO205, HT‐29, and RG/C	[88]
LGR5 + stem cells uniquely respond to alkylation-induced DNA damage by upregulating DNA damage repair, apoptosis and cell proliferation	Colorectal	LGR5-EGFP-IRES-creERT2 knock-in mice with and without colon cancer	[89]
Knockdown of *LGR5* induced apoptosis	Colorectal	Colon cancer cell line: SW620	[90]
LGR5 increased tumorigenicity via upregulating WNT/ β-catenin pathway	Colorectal	CRC tissues (*n* = 74)	[46]
LGR5-KD reduced the survival of cancer cells, induced proliferation, and enhanced cell cycle progression	Colorectal	CRC cell line: RG/C2	[91]
Ablation of LGR5-positive cells inhibited growth of CRCs *in vitro* and *in vivo*	Colorectal	Human colon epithelial adenocarcinoma cell lines: HT-29, DLD-1, Caco-2, and LS123 NOD/SCID mice with Caco-2 cells	[92]
*LGR5*-positivity is associated with cancer colony formation, self-renewal, differentiation, and tumorigenicity	Colorectal	CRC cell line DLD-1 (CCL-221)	[93]
Overexpression of LGR5 inhibited in the proliferation of CRCs	Colorectal	Human CRC cell lines: DLD-1, HT29, SW620, HCT116, HCT15, LOVO, SW480, KM12C, KM12L4, and KM12SM	[60]
LGR5 overexpression promoted cell growth and colony formation. LGR5-KD reduced cell proliferation and colony formation	Endometrial	Endometrial cancer cell lines: AN3CA and Ishikawa	[94]
High *LGR5* expression was significantly correlated with proliferation via regulation of WNT and EMT	Oral squamous cell carcinomas (OSCC)	OSCC patients (*n* = 78)	[63]
LGR5 over-expression promotes gastric cancer cell proliferation and sphere cell growth LGR5 enhanced tumorigenesis *in vivo* by increasing spheroid sizes	Gastric	Human gastric adenocarcinoma cell lines: MGC803 Female BALB/c nude mice	[95]
LGR5 + enhanced tumoregenesis *in vitro* and *in vivo*	Gastric	Human gastric adenocarcinoma cell lines: MKN45 and MKN28 Female athymic BALB/c nu/nu mice	[96]
*LGR5* is involved in WNT-driven tumorigenesis in the stomach	Gastric	Gastric cancer patients (*n* = 159)	[97]
Ablation of *LGR5*-positive cells inhibited growth of gastric cancer cells *in vitro* and *in vivo*	Gastric	Human gastric cancer cell line: SNU-5 NOD/SCID mice	[98]
LGR5 overexpression increased spheroid forming, canonical R-SPO1/WNT3a signaling and cell proliferation	Hepatic	Hepatic cancer cell lines: HuH-7, Hep3B; mesenchymal-like cell lines: SNU-449	[99]
Overexpression of *LGR5* promoted HCC cell viability and enhanced colony formation	Hepatic	HCC cell lines: KY-G1 and KY-V2	[100]
LGR5 promoted tumor proliferation and clone formation	Lung	NSCLC tissues (*n* = 22) Lung cancer cell lines: A549 and H1299	[13]
Knockdown of *LGR5* induced WNT-independent apoptosis	Neuroblastoma	Neuroblastoma cell lines: SK-N-BE(2)-C, SK-N-AS and SH-SY5Y	[101]
Silencing of LGR5 inhibited cell proliferation	Oesophageal	ESCC cell lines: KYSE70, Eca9706, Eca109, and KYSE450	[39]
LGR5 promoted proliferation *in vitro* LGR5 promoted tumorigenesis* in vivo*	Ovarian	Ovarian cancer cell line: SKOV3, Hey (LGR5 silenced) and HO8910 (LGR5 over-expressed) Female BALB/c athymic nude mice with SKOV3 OC cells (*n* = 6)	[38]
LGR5 co-expressed with Ki67 in HGSOC	Ovarian	Ovarian cancer (*n* = 50): benign tumor (*n* = 14), borderline tumor (*n* = 14), high-grade serous? metastatic tumor (*n* = 14), normal ovarian tissue (*n* = 8)	[2]
Reduced proliferation was observed in cells transfected with LGR5	Ovarian	Ovarian cancer cell lines: OVCAR-3 and SNU-8	[61]
Adhesion			
LGR5 depletion led to a downregulation of L1 cell adhesion molecule expression	Brain	Brain CSC lines NCH421k and NCH441 cells	[73]
Overexpression of LGR5 altered the actin cytoskeleton and increased cell–cell adhesion	Colorectal	Colon cancer cell line: CHO	[102]
Overexpression of LGR5 increased cell adhesion	Colorectal	Human colorectal cancer cell line: LIM1899	[77]
Overexpression of LGR5 increased cell adhesion	Colorectal	Patient-derived colorectal cancer organoids with LGR5 + labels	[103]
Migration & Invasion			
LGR5 promoted cell migration and EMT via the WNT/ β-catenin pathway	Breast	Breast cancer cell line: MCF-7	[74]
Elevated LGR5 expression in cervical cancer cells increased cell migration and invasion	Cervical	Human cervical carcinoma cell lines HeLa and SiHa	[58]
CASC15 overexpression promoted colon cancer cell migration and invasion through activation of WNT/β-catenin pathway by upregulating LGR5 expression through microRNA-4310	Colorectal	Human colon cancer cell lines: HT29, HCT116, SW480, SW620	[85]
*LGR5* silencing significantly reduced migration	Colorectal	Colon cancer cell line: CT-26	[82]
LGR5-KD reduced the invasive ability of HT29 *in vitro*	Colorectal	Human colon cancer cell lines: HT-29	[80]
LGR5 silencing reduced migration *in vitro*	Colorectal	CRC cell lines: Caco-2, HCT 116, HT-29, SW480, SW620 and T84	[75]
LGR5 was positively correlated with depth of invasion	Colorectal	CRC tissues (*n* = 192)	[86]
LGR5 promoted invasion of HCT116 spheroids via EMT	Colorectal	Human colon cancer cell lines: HCT116	[83]
Ablation of LGR5 increased invasion	Colorectal	Colon cancer cell line: LIM1899	[77]
LGR5 protein expression in CRC tissues was significantly associated with vascular invasion	Colorectal	CRC tissues (*n* = 53)	[76]
High LGR5 expression was associated with increased tumor invasion	Colorectal	Primary CRC tissues (*n* = 51)	[104]
High expression of LGR5 was significantly associated with vascular invasion	Colorectal	CRC tissues (*n* = 89)	[49]
LGR5 protein expression significantly correlated with the depth of tumor invasion	Colorectal	CRC tissues (*n* = 120 males, *n* = 84 females)	[105]
LGR5 and CD133 co-expression was positively correlated with invasion	Colorectal	CRC tissues (*n* = 100)	[106]
LGR5 + cells had increased vascular infiltration	Colorectal	CRC tissues (*n* = 89)	[107]
No association was found between LGR5 expression and invasion	Colorectal	CRC tissues (*n* = 21 males and *n* = 19 females)	[108]
Knockdown of LGR5 suppressed cell migration	Colorectal	Human CRC cell lines: DLD-1, HT29, SW620, HCT116, HCT15, LOVO, SW480, KM12C, KM12L4, and KM12SM	[60]
LGR5 over-expression promotes gastric cancer cell migration	Gastric	Human gastric adenocarcinoma cell lines: MGC803	[95]
Enhanced LGR5 expression was not associated with lymph vascular invasion or depth of invasion	Gastric	Intestinal metaplasia (*n* = 90), dysplasia (*n* = 53), gastric adenocarcinoma (*n* = 180)	[42]
Overexpression of *LGR5* decreased cell motility	Hepatic	HCC cell lines: KY-G1 and KY-V2	[100]
LGR5 overexpression enhanced cell migration and invasion	Hepatic	Hepatic cancer cell lines: HuH-7-mock and HuH-7-LGR5-OE	[99]
LGR5 expression was positively associated with tumor invasion	Lung	Primary NSCLC (*n* = 122)	[109]
LGR5 expression enhanced tumor migration and invasion	Lung	Lung cancer cell lines: A549 and H1299 (Mock- or LGR5- transfected)	[13]
Silencing of *LGR5* inhibited migration and invasion of ESCC cells	Oesophageal	ESCC cell lines: KYSE70, Eca9706, Eca109, and KYSE450	[39]
LGR5 facilitated migration and invasion of EOC cells *in vitro*	Ovarian	Ovarian cancer cell line: SKOV3, Hey (LGR5 silenced) and HO8910 (LGR5 over-expressed)	[38]
LGR5 induction decreased tumor migration	Ovarian	Ovarian cancer cell lines: OVCAR-3 and SNU-8	[61]
High expression of LGR5 correlated with increased depth of tumor invasion	Rectal	Rectal cancer tissues (*n* = 30)	[12]
High LGR5 and RSPO2 mRNA and protein expression were associated with increased vascular invasion, capsular invasion, and macroscopic invasion	Thyroid	Thyroid cancer cell lines (TPC-1, KTC-1, Nthy-ori-3–1) (*n* = 3) Thyroid cancer tissues (*n* = 26)	[110]
Metastasis & Angiogenesis			
LGR5 expression promotes tumor metastasis	Breast	Breast cancer cell line: MCF-7	[74]
High levels of *LGR5* were significantly associated with LN metastasis	Breast	Breast cancer tissues (*n* = 126)	[111]
Ablation of LGR5 + cells reduced colon-cancer-derived liver metastatic burden	Colorectal	Apc^min/+^, Kras^LSL−G12D/+^, Vil1^Cre^ (AKV) were crossed with LGR5^DTR/eGFP^ mice (*n* = 4)	[45]
LGR5 suppressed metastasis of colon cancer cells *in vivo*	Colorectal	Xenografts model with male athymic nude mice	[84]
LGR5- cells had an increased ability to migrate into the surrounding stroma and portal venous circulation or seed liver metastasis compared to LGR5 + cells	Colorectal	*In vivo*: male and/or female NOD. Cg-Prkdc^scid^II2rg^tm1WjI^/Szj (NSG) mice	[112]
Lower LGR5 expression was significantly correlated with the presence of LN metastases	Colorectal	CRC tissues (*n* = 89)	[49]
No association was found between regional LN metastasis and LGR5 expression	Colorectal	CRC tissues (*n* = 21 males and *n* = 19 females)	[108]
LGR5 + cells had a 6–11.5-fold higher metastasis level than cells with no LGR5 expression	Colorectal	CRC tissues (*n* = 89)	[107]
LGR5 was positively correlated with LN metastasis and distant metastasis	Colorectal	CRC tissues (*n* = 192)	[86]
LGR5 expression correlated with metastasis	Colorectal	CRC patients’ tissues (Stages I-IV n = 54, Controls n = 19)	[111]
Positive LGR5 protein expression was significantly correlated to LN metastasis	Colorectal	CRC tissues (*n* = 120 males, *n* = 84 females)	[105]
LGR5 and CD133 co-expression was positively correlated with LN metastasis and distant metastasis	Colorectal	CRC tissues (*n* = 100)	[106]
High *LGR5* expression level significantly correlated with the occurrence of metastasis	Colorectal	CRC tissues (*n* = 66)	[111]
High LGR5 expression is associated with distant metastasis	Colorectal	CRC tissues from patients with chemotherapy treatment (*n* = 64)	[40]
LGR5 protein expression in CRC tissues was significantly associated with LN metastasis and tumor angiogenesis	Colorectal	CRC tissues (*n* = 53)	[76]
LGR5 over-expression was more frequently found in the metastatic tissues of both LN and distant areas compared with primary CRC tissue	Colorectal	Matched primary and normal CRC tissues (*n* = 51)	[104]
*LGR5* mRNA expression level was significantly higher in the tissue with LN metastasis than those tissues without metastasis	Colorectal	CRC tissues (*n* = 32), normal colon tissues (*n* = 32). CT-26 cell line	[82]
Higher expression of *LGR5Δ5* variant (LGR5 lacking exon 5) was associated with LN metastases	OSCC	OSCC tissues (*n* = 78)	[63]
LGR5 in primary tumors significantly correlated with LN metastasis	Gastric	Gastric cancer tissues (*n* = 119)	[113]
*LGR5* siRNA enhanced angiogenesis by increasing VEGF expression level	Gastric	Human gastric cancer cell line: AGS (silencing genes: LGR5-homo-409, LGR5-homo-1555 and LGR5-homo-2664)	[114]
Enhanced LGR5 protein expression was not associated with LN metastasis or distant metastasis	Gastric	Gastric adenocarcinoma tissues (*n* = 180)	[42]
LGR5 expression was positively associated with metastasis	Lung	Primary NSCLC tissues (*n* = 122)	[109]
LGR5 expression was associated with LN metastasis	Lung	NSCLC tissues (*n* = 22)	[13]
LGR5 expression significantly correlated with LN metastasis	Oesophageal	ESCC tissues (*n* = 280)	[39]
LGR5 expression positively correlated to LN metastasis	OSCC	OSCC tissues (*n* = 190)	[115]
LGR5 facilitated metastasis in EOC cells *in vitro*	Ovarian	Ovarian cancer cell line: SKOV3, Hey (LGR5 silenced) and HO8910 (LGR5 over-expressed)	[38]
A positive association between LGR5 expression, metastasis marker (vasohibin-1) and lymph node metastasis	Ovarian	EOC patient tissues (*n* = 210)	[48]
High LGR5 expression correlated with LN metastasis	Rectal	Rectal cancer tissues (*n* = 30)	[12]
High LGR5 mRNA and protein expression were associated with LN metastases	Thyroid	Thyroid cancer tissues (*n* = 26)	[110]

Abbreviation: Epithelial ovarian cancer (EOC), knockdown (KD), overall survival (OS), epithelial-mesenchymal transition (EMT), lymph node (LN), non-small cell lung cancers (NSCLC), oesophageal squamous cell carcinoma (ESCC), colorectal cancer (CRC), and oral squamous cell carcinomas (OSCC), protein kinase A (PKA), cancer susceptibility 15 (CASC15), vascular endothelial growth factor (VEGF), specificity protein 1 (SP1)

### LGR5 in tumor proliferation and tumorigenesis

High LGR5 expression enhanced tumor proliferation through WNT/β-catenin in lung [13], gastric [95], CRC [40, 63, 75, 76, 78–87, 89, 91], hepatocellular carcinoma (HCC) [99, 100], breast [57, 74], cervical [41, 58], endometrial [94], oesophageal [39], bone [43], and brain [71–73] cancers. In CRC cell lines (LOVO, HT29, HCT116), LGR5-KD suppressed cell proliferation and colony formation [48, 60] and LGR5 overexpression enhanced cell proliferation [40]. LGR5-KD reduced WNT/β-catenin pathway activation and LGR5 overexpression led to elevated TCF/LEF activity and induced WNT/ β-catenin pathway in HCC cells (HuH-7) [99]. LGR5-KD in ovarian cancer (HO8910) cells significantly inhibited cell proliferation [38]. Lower levels of cyclin D1 and C-Myc were also observed in LGR5-KD SKOV3 cells compared to parental cells, suggesting that LGR5 can promote cell proliferation, cell growth and cancer metastasis in epithelial ovarian cancer [38]. LGR5 protein expression was positively correlated with cervical cancer proliferation *in vitro* and *in vivo* [41]. Silencing of LGR5 expression suppressed brain tumor cell proliferation and cell cycle *in vitro* [72]. LGR5 overexpression promoted the formation and proliferation of CRC spheroids [83]. LGR5+ depletion suppressed CRC growth and LGR5+ cells could re-initiate tumor growth *in vivo* [45].

Studies in gastric [97, 98], colorectal [45, 75, 92, 93], breast [70, 74] and brain [72] cancer found that LGR5 overexpression enhanced tumorigenesis *in vivo*. LGR5+ gastric tumor cells were able to generate tumors in primary, secondary, and tertiary transplantation, whereas cells from LGR5^−^ tumors lost tumorigenic potential during serial transplantations *in vivo* [96]. *LGR5* has also been shown to be involved in CRC tumorigenesis and plays a role as a pro-oncogene, promoting the WNT/β-catenin signaling pathway [46]. A significant positive correlation between LGR5 and Ki-67 (proliferation marker) expression was observed in the CRC tumor tissues (r^2^=0.680, *p*<0.001) [46, 86, 91]. LGR5 silencing also reduced tumorigenesis *in vivo* using CRC [75, 80] and ovarian cancer models [38] .

Knockdown of *LGR5* induced apoptosis in colon [40, 79, 80, 88, 90], breast [57], and neural [73, 101] cancers. *LGR5* depletion-induced apoptosis was associated with a loss of mitochondrial membrane potential in CRC cells [79]. *LGR5* depletion also induced apoptosis of neuroblastoma cells independent of WNT/β-catenin signaling pathway [101]. To date limited studies have investigated the role of LGR5 on tumor apoptosis highlighting a need for future research.

LGR5 enhanced clonogenicity in many cancers, including colon [40, 75, 84, 93], hepatic [100], breast [70, 74] and ovarian [38] cancers proliferation. Overexpression of *LGR5* promoted HCC cell viability and enhanced colony formation [100].

Although the majority of cancer studies suggest that LGR5 promotes tumor growth, several studies have reported the opposite results [60, 61, 77, 91, 110]. Studies investigating the role of LGR5 in early colorectal adenomas found that LGR5-KD reduced the survival of epidermal growth factor (EGF)-treated cancer cells, induced proliferation, and enhanced cell cycle progression [91]. However, EGF treatment which can stimulate cell growth, proliferation, and differentiation, complicated this study because both the EGFR and WNT/β-catenin pathways would be activated in these cells. Overexpression of LGR5 inhibited proliferation and colony-formation of CRCs cells (DLD1) [60]. LGR5 knockdown increased the clonogenicity of both LIM1215 and LIM1899 CRC cells [77]. An ovarian cancer study also found that LGR5 overexpression suppressed cell proliferation and progression of a high grade ovarian cancer cell line (OVCAR-3) 95. Together these results indicate that LGR5 play either a suppressive or promoting role in tumor growth and effects may be cell-dependent. Additional studies are required to carefully dissect the differences between the studies that reported LGR5 anti-tumorigenic effects and studies that reported LGR5 enhances tumorigenesis. The functional effect of LGR5 on tumor growth may be dependent on cancer type, TME and cross-talk with other molecules in LGR5 signaling pathway.

### LGR5 in cancer cell adhesion, tumor migration and invasion

To date, studies investigating LGR5 in cancer cell adhesion and migration are limited. LGR5 enhanced cell–cell adhesion in stem cells of CRC via the IQGAP1–Rac1 (GTPase-activating-like protein) pathway [102]. Overexpression of LGR5 could augment CRC cell adhesion [77, 103]. LGR5 depletion downregulated brain cancer cell adhesion [73]. High LGR5 expression has been shown to be associated with increased tumor migration in cancers including gastric [95], CRC [60, 75, 82], breast [74], and HCC [99] cancers. LGR5 was co-expressed with EMT markers (E-cadherin and β-catenin) and EMT inducers (PRRX1, TWIST1, and BMI1) in gastric cancer spheroids [95]. LGR5 promoted breast cancer cells to undergo EMT through the WNT/β-catenin signaling pathway [74]. Interestingly, enhanced WNT signaling was also detected in CSCs due to increased β-catenin localization [116], suggesting that LGR5 is important in restricting stem cells to their niche. A gastric cancer study suggested that when the LGR5 + stem cell population is depleted, LGR5- cells move into the stem cell niche and are directed to revert to LGR5 + stem cells, thereby restoring tissue homeostasis [112]. However, one study also showed that overexpression of LGR5 decreased HCC (KY-V2) cell motility [100]. This study also showed that LGR5-KD increased cell motility in an LGR5-transfected clone with LGR5 overexpression [100]. A limitation of this study is that they only used one HCC cell line.

High LGR5 expression enhanced tumor invasion in cervical [58], rectal [12], CRC [49, 76, 77, 80, 83, 85, 86, 104–108], lung [13, 109], ESCC (oesophageal squamous cell carcinoma) [39], thyroid [110], and hepatic [99] cancers. In a CRC study, LGR5 protein expression positively correlated with vascular invasion (CD34 expression) [76]. However, another gastric cancer study found a correlation between LGR5 expression and tumor stage but no relationship with lymph node invasion [42]. However, a limitation of this study is that they only measured LGR5 expression in a small number of lymph node tissues.

To date, only two studies investigated the role of LGR5 in ovarian cancer migration or invasion. LGR5-KD SKOV3 cells had reduced wound healing ability *in vitro*, reduced expression of mesenchymal markers (N-cadherin and vimentin), and enhanced expression of the epithelial marker (E-cadherin) [38]. However, LGR5 overexpression was associated with reduced migration in ovarian cancer cells (OVCAR-3 and SNU-8) [61]. These contradictory findings may be due to the different ovarian cancer cells used in the studies and further studies are warranted to corroborate these findings.

The majority of cancer studies have shown that LGR5 was associated with WNT/β-catenin signaling, which increases the expression of target genes like E-cadherin (cell adhesion marker) via EMT, enhancing tumor adhesion. Moreover, LGR5 enhances the expression of transcription factors such as Snail, Slug, and Twist, via EMT, which are involved in suppressing epithelial characteristics and promoting a mesenchymal, invasive phenotype [17], increasing tumor migration and invasion ability of cancer cells. Further studies are warranted to address the discrepancies in the role of LGR5 in cancer migration and tumor invasion.

### LGR5 in angiogenesis

To date, limited studies have investigated the relationship between LGR5 and angiogenesis in cancers. High LGR5 expression was associated with enhanced angiogenesis in gastric [114] and CRC tissues [76]. High LGR5 expression in ovarian cancer was found to be associated with high vasohibin-1 (angiogenesis inhibiting protein-1, infiltration & metastasis marker) expression, a protein associated with early infiltration and metastasis in many cancers [48]. LGR5 has been linked to angiogenesis through its role in promoting CSC, secreting angiogenic factors like VEGF, and interacting with the TME, all of which contribute to tumor vascularization and progression [114, 117]. However, the involvement of LGR5 + cells in TGFβ pathway makes it more complex, and further research is needed to clarify these dynamic interactions.

### LGR5 in metastasis

Overexpression of LGR5 is associated with metastasis in CRC [12, 40, 45, 76, 82, 86, 104–107, 111, 118], breast [63, 74, 119, 115], ESCC [39], lung [13, 109], gastric [113], thyroid [110], and ovarian [38] cancer. LGR5 was positively correlated with distant metastasis and poor clinical outcome in CRC and gastric cancer [86, 107, 118]. LGR5-KD significantly reduced the metastasis of breast cancer (MDA-MB231) cells *in vivo* [74]. Interestingly, low LGR5 protein expression has also been shown to be associated with increased lymph node metastases in CRC tissues [49]. The authors suggested that downregulation of LGR5 expression may promote EMT and increase metastatic ability of CSC [49]. This hypothesis may explain why lower LGR5 expression is associated with lymph node metastases. Another *in vivo* CRC study showed that LGR5 suppressed metastasis as only 29% of control group mice had lung or liver metastasis compared to 77% of LGR5-KD mice which had lung or liver metastasis [84]. This *in vivo* study found no enhancement of WNT signaling by LGR5. Instead, LGR5 interacted with TGFβ signaling pathways, triggering Smad activation to suppress tumor metastasis. These findings highlight that LGR5 has a context-dependent role, depending on different TME, LGR5 may promote metastasis or activate tumor-suppressive pathways. Moreover, two CRC studies found no association between LGR5 protein expression and distant metastasis [40, 108]. A study in gastric cancer also found no relationship between LGR5 expression and metastasis [42]. Another *in vivo* CRC study selectively depleted the LGR5 + stem cell population also showed that LGR5- cells drive tumor metastasis and migration [120]. Using mouse models, this study showed that LGR5- cells can revert to a stem-cell-like state under certain conditions, particularly during the metastatic process [120]. This research highlights that LGR5- cells exhibit significant plasticity and contribute to cancer progression and metastasis, suggesting that both LGR5 + and LGR5- cells should be targeted when developing cancer treatment. Together these findings show that the relationship between LGR5 and metastasis is complex and LGR5 may play context-dependent roles. Depending on different TMEs, LGR5 may promote metastasis or activate tumor-suppressive pathways.

LGR5+ cells are stem-cell-like and can initiate tumor growth and secondary tumors in distant sites. LGR5+ cells enhanced WNT/β-catenin pathway and detachment of tumor cells which migrate to nearby lymph nodes. However, LGR5+ can interact with TGFβ, suppressing tumor metastasis in early-stage tumors. The TGFβ pathway has a dual role in cancer [121], in advanced cancer, the TGFβ pathway promotes tumor progression by enhancing EMT and tumor invasion. In early-stage cancer, TGFβ inhibits cell proliferation, induces apoptosis, and maintains tissue homeostasis by promoting differentiation and reducing inflammation [121]. LGR5, as a receptor involved in WNT signaling, may facilitate the controlled activation of this pathway, ensuring that it supports tissue maintenance and repair while preventing its excessive activation, which could drive tumorigenesis [122]. By maintaining balanced WNT/β-catenin signaling, LGR5 helps preserve normal tissue architecture and curtails aberrant cellular proliferation that can lead to cancer progression. Further investigation is required to address the disparate and conflicting findings to increase our understanding on the role of LGR5 in tumor metastasis.

## Role of LGR5 chemotherapy resistance

Several studies have shown that LGR5 expression is increased following the development of chemotherapy resistance. A potential reason why LGR5 is associated with chemoresistance is that LGR5 is a key receptor in WNT/β-catenin signaling pathway which plays a critical role in maintaining the stemness of CSCs [10]. Activation of this pathway enhances the survival and proliferation of LGR5 + cells, contributing to their resistance to chemotherapeutic agents [10]. Table 2 summarizes the studies that have investigated the role of LGR5 in chemotherapy resistance in different cancers including breast, cervical, colorectal, gastric, hepatocellular and ovarian cancer.

**Table 2:** Role of LGR5 in chemotherapy resistance

Major Findings	Tumor Type	Cell lines/Animal species	Ref
LGR5 increases the aggressiveness and cisplatin resistance of breast cancer cells through PKA	Breast	MCF-7	[123]
*LGR5-KD* inhibited docetaxel drug resistance	Breast	Breast cancer cell line: MCF-7, MDA-MB-231, BT-549, ZR-75–1	[57]
Elevated LGR5 expression in cervical cancer cells conferred chemo-resistance to cisplatin treatment	Cervical	Human cervical carcinoma cell lines HeLa and SiHa Female NOD/SCID mice	[58]
*LGR5-KD* enhanced chemo-sensitivity (5-FU or cisplatin) *in vitro*	Colon	HT-29 spheroids BALB/C-nude mice	[80]
Increased *LGR5* expression observed in *PIK3CA* mutation chemoresistant tumor cells is associated with shorter OS	Colon	Colon cancer patient tissues (*n* = 440)	[52]
Peritoneal recurrence was significantly higher in LGR5-negative cases	Colon	Primary non-metastatic colon cancer tissues (*n* = 292)	[51]
HEK293/LGR5 cells were 1.6-fold more resistant to free MMAE (Monomethyl auristatin E) than the HEK293/EV cells	Colon	HEK293 cells	[53]
Patients with high LGR5 expression responded better to chemotherapy treatment than those with low LGR5 expression *LGR5-KD* rendered cells are more sensitive to chemotherapeutic agents (oxaliplatin or 5-FU)	Colorectal	CRC tissues from patients with chemotherapy treatment (*n* = 64)	[40]
LGR5- cells in colon cancer cells enhanced resistance to irinotecan and 5-FU	Colorectal	CRC cell lines: DLD-1, HT-29, LS180 and LOVO	[124]
miR-132 promoted cisplatin resistance in LGR5 + GCSCs *in vitro* and *in vivo*	Gastric	Cell lines: MKN45 and MKN28 Female athymic BALB/c nu/nu mice	[96]
LGR5 over-expression induced oxaliplatin resistance	Gastric	Human gastric adenocarcinoma cell lines MGC803	[95]
*LGR5-KD* resensitized DAP3 (Death Associated Protein 3)-depleted gastric cancer cells to 5-FU and oxaliplatin	Gastric	Human gastric adenocarcinoma cell lines HGC27 and MGC803 cells	[120]
Inhibition of *LGR5* increased the sensitivity of AGS gastric cancer cells to chemotherapy (oxaliplatin-based)	Gastric	Gastric cancer cell line: AGS	[125]
LGR5 + from primary tissues and cell lines are resistant to doxorubicin	Hepato-cellular	HCC cell lines: PLC9024, Huh7 spheroids	[126]
Higher *LGR5* expression is associated with carboplatin resistance	Ovarian	SKOV3	[127, 62]
Ovarian cancer-programmed into iPSCs cells highly expressed LGR5 were more resistant to cisplatin and taxol	Ovarian	Wild type PEO4 cells PEO4-iPSC-OSKM-05	[128]

Abbreviation: gastric cancer stem cell-like (GCSCs), 5-Fluorouracil (5-FU), colorectal cancer (CRC), overall survival (OS) protein kinase A (PKA).

Breast and cervical cancer studies reported that high LGR5 expression was associated with chemotherapy resistance. LGR5 promoted cisplatin resistance of breast cancer cells (MCF-7) through PKA (Protein Kinase A) [123] and *LGR5-KD* inhibited docetaxel drug resistance [57]. A cervical cancer study found that LGR5 promoted CSCs traits and chemo-resistance through the WNT/β-catenin signaling pathway [58]. Overexpression of LGR5 enhanced chemo-resistance in CRC cells whilst LGR5-KD cells were more sensitive to 5-FU chemotherapy [40]. Furthermore, LGR5-KD in CRC spheroids increased chemo-sensitivity to cisplatin, oxaliplatin and 5-FU [40, 80]. LGR5 marks a subpopulation of CSCs that are inherently more resistant to chemotherapy. LGR5 + CSCs have slow cell cycles and can remain dormant, allowing them to evade therapies that target rapidly dividing cells [84]. After chemotherapy, these cells can survive and regenerate the tumor, leading to recurrence [84].

Several gastric cancer studies have indicated relationships between LGR5 expression and chemotherapy resistance. LGR5 overexpression was associated with oxaliplatin resistance in gastric MGC803 cells *in vivo* [95]. Another gastric cancer study found that LGR5-KD could re-sensitize DAP3-depleted gastric cancer cells to 5-fluorouracil (5-FU) and oxaliplatin [120]. LGR5 + gastric cancer stem cell-like cells (GCSCs) were associated with cisplatin resistance [120]. In this study, miR-132 (tumor progression marker) expression was enhanced in LGR5 + GCSCs and correlated with chemo-resistance in gastric cancer patients [120]. The same study also observed that LGR5 promoted chemoresistance by upregulating multidrug resistance protein 1 (MDR1, also known as ABCB1 or p-glycoprotein) [120]. A HCC study reported that LGR5 + cells were able to form compact self-renewing spheres associated with doxorubicin resistance, while LGR5- cells could not form tumor spheroids [126]. High expression of LGR5 in ovarian cancer cells was associated with resistance to carboplatin [62, 127]. PE04 ovarian cancer cells programmed into pluripotent stem cells (iPSCs) expressed high levels of LGR5 and were more resistant to cisplatin and taxol compared to parental PEO4 cells [128].

LGR5 + cells can interact with TME by recruiting immune cells and secreting factors that can enhance angiogenesis or inhibit immune responses, creating a niche where CRC cells can survive from chemotherapy or other treatments [84]. LGR5 plays a role in cell plasticity that has also been associated with chemotherapy resistance. For example, a recent CRC study suggested that LGR5 maintains its CSC properties and contributes to cell plasticity, enhancing cancer cell transition between differentiated and stem-like states [124]. Zhang *et. al.* (2019) found that LGR5 + CSCs can transition between LGR5- cancer cells, and LGR5- cells have been shown to exhibit greater resistance to both chemotherapy and radiotherapy [124]. The mechanisms driving the plasticity of LGR5- cancer cells remain poorly understood and require further research.

In summary, many studies have found that high LGR5 expression is associated with chemoresistance in breast, cervical, colorectal, hepatic, gastric and ovarian cancer to a wide range of chemotherapy drugs including carboplatin, cisplatin, paclitaxel oxaliplatin, 5-FU and doxorubicin. LGR5 is associated with chemoresistance due to its role in maintaining CSCs, promoting tumor survival pathways, driving drug efflux mechanisms, and enabling interactions with the TME. Overcoming chemoresistance in cancers with high LGR5 expression likely requires targeting both LGR5 + and LGR5- cells.

## Strategies for targeting LGR5 in cancers

LGR5 is involved in promoting tumor progression via EMT and WNT/β-catenin pathways and increased LGR5 expression is associated with chemotherapy resistance and recurrence in different cancers including breast, cervical, CRC, gastric, HCC and ovarian carcinoma. LGR5 is a potential target for therapeutic strategies aimed at overcoming chemoresistance in these cancers. Studies to date investigating LGR5 inhibitors or LGR5 targeting strategies in cancers are summarised in Table 3.

**Table 3:** LGR5 inhibitors and drugs targeting the LGR5 protein in cancers

Major Findings	Antibody/Inhibitor	Cell line/Animal model	Ref
4-AAQB treatment reduced LGR5 protein expression by western blot	4-AAQB	CRC cell line HT29, HCT116	[129]
Both anti-LGR5 conjugates (10 mg/kg) resulted in tumor stasis or tumor regression in both LOVO and D5124 *in vivo* models Continuously targeting LGR5 + cells with anti-LGR5–vc-MMAE can have long-term effects on reduced tumor growth and extended overall survival *in vivo*	Antibody drug conjugate: Anti-LGR5–vc-MMAE and anti-LGR5–NMS818	Immunodeficient mice with colon cancers: LOVO and D5124 xenografts Vil1^Cre^ (AKV) model (*n* = 37)	[130]
BNC101 was tested in a safety and dose escalation phase I clinical trial in patients with recurrent metastatic CRC BNC101 targeted LGR5 and co-localized with LGR5 in CRC patient tissues	BNC101	Clinical trial	[131]
Curcumin (5 μM) inhibited tumor-sphere formation, decreased cell viability and promoted apoptosis of LGR5( +) colorectal CSCs and suppressed LGR5( +) colorectal CSCs	Curcumin	CRC cell line SW620	[132]
LGR5-targeting CAR-T cells showed significant antigen-specific cytotoxicity against CRC cell lines *in vitro* LGR5-targeting CAR-T cells were able to inhibit the growth of human CRC tumors significantly *in vivo*	LGR5-CAR-T	CRC cell line: LOVO	
LGR5-CAR-T cells were cytotoxic, exhibited anti-tumor activity and significantly inhibited the survival of OC cell lines (COV318, COV362), primary HGSOC ovarian cancer cells *in vitro*	LGR5-CAR-T	Ovarian cancer cell lines: COV318, COV362, primary HGSOC	[133]
NEDD4 and NEDD4 ligand targeted LGR5 receptor for lysosomal and proteasomal degradation	NEDD4 and NEDD4 ligand	Human embryonic kidney cell line: HEK293	[134]
Doxorubicin carried by the RSPO1-liposomes was more effective at lower concentrations targeting LGR5 + cells. Massive tumor tissue necrosis and growth inhibition were observed	RSPO1-Doxorubicin	CRC cell lines: LOVO and RAW264.7 Female BALB/c nude mice. GA007 PDX tumor tissues (*n* = 3)	[135]
TCS inhibited the expression of LGR5 and key proteins in the Wnt/β‑catenin signaling pathway in glioma cells	TCS	Human malignant glioma cell lines U87	[136]
VJ treatment in ovarian cancer cells reduced LGR5 expression	VJ	A2780, A2780/CP7, OVCAR5	[137]
VJ treatment in lung cancer cells reduced *LGR5* expression, induced apoptosis, and inhibited WNT1/β-catenin and Notch1 pathways	VJ	Lung cancer A549 cell line	[138]

Abbreviation: 4-Acetylantroquinonol B (4-AAQB), chimeric antigen receptor T (CAR-T), colorectal cancer (CRC), High grade serous ovarian carcinoma (HGSOC), Neuronal precursor cells developmentally downregulated protein 4 (NEDD4), Patient derived xenograft (PDX), R-spondin 1 (RSPO1), Trichosanthin (TCS), Verrucarin J (VJ)

4-Acetylantroquinonol B (4-AAQB) is a bioactive isolate from a Taiwanese mushroom with documented anti-inflammation, hypoglycaemic, vasorelaxant, and recently demonstrated antiproliferative activity [129]. 4-AAQB negatively regulated stem cell maintenance via the LGR5/WNT/β-catenin signaling pathway. LGR5 expression was dramatically reduced following treatment with either 5µM or 10µM 4-AAQB in colon cancer cells (HT29, HCT116) and inhibited tumor growth *in vivo* [129]. Further studies using 4-AAQB in other cancers are warranted to confirm these findings and evaluate its potential to target LGR5.

Curcumin can suppress oncogenicity in many cancer cells [132]. A CRC study used curcumin to target LGR5+ CSC and significantly suppressed CSCs formation *in vitro* [132]. Trichosanthin (TCS), a bioactive protein extracted and purified from the tuberous root of *Trichosanthes kirilowii* (a well‑known traditional Chinese medicinal plant), induced apoptosis also by targeting LGR5 and repressing the WNT/β‑catenin signaling pathway in glioma cells [136]. LGR5 protein expression was decreased in glioblastoma cancer cells (U87) when treated with increasing concentrations of TCS *in vitro *[136].

Verrucarin J (VJ) is a type D macrocyclic sesquiterpenoid mycotoxin. It is a potent anticancer drug and suppresses LGR5 expression and ovarian cancer tumor growth and metastasis [137]. In the A2780 ovarian cancer xenograft model, mice treated with 0.1 mg/kg VJ and 0.5 mg/kg VJ had a 32% and 61% significant reduction in tumor weight compared to the control group, respectively [137]. Another study using a lung cancer cell line (A549) found that VJ significantly inhibited both Notch1 and WNT/β-catenin pathways [138]. This study found that VJ suppressed LGR5 protein expression by inhibiting CSC self-renewal signaling pathways [138]. VJ has potent anticancer activity and can target LGR5 in different cancer cells and CSCs. It will be important to assess VJ further *in vivo* studies to verify these findings.

Anti-LGR5 antibody-drug conjugates (ADCs) have also been found to be effective in treating tumors with high expression of LGR5. Two ADCs targeting LGR5+ CRC cells had significantly increased survival in mice *in vivo* [130]*.* Anti-LGR5-ADC effectively inhibited tumor growth in MDA-MB231 and patient-derived xenografts with high-LGR5 in breast cancer [70]*.* A humanized monoclonal LGR5 antibody - BNC101 targeting LGR5 in tumor biopsies was evaluated in safety and dose escalation phase I clinical trials in patients with recurrent metastatic CRC [131]. Although the trial was terminated, there was evidence that BNC101 could infiltrate the CRC patient tumor and engage with overexpressed LGR5 receptors [131]. A recent study found a combination treatment with oxaliplatin-loaded magnetoliposomes functionalized with LGR5 antibody reduced the proliferation of CRC cell lines compared to free drug [139].

Neuronal precursor cell developmentally downregulated protein 4 (NEDD4) which belongs to the NEDD4 family of HECT-type E3 ubiquitin ligases, a proto-oncogene targeting the tumor suppressor PTEN (Phosphatase and TENsin homolog deleted on chromosome 10) involved in lysosomal and proteasomal degradation in kidney cancer cells has been shown to target LGR5 *in vitro*. The study showed that NEDD4 targeting LGR5, negatively regulated WNT signaling upstream of the β-catenin destruction complex and loss of NEDD4 increased the sensitivity to RSPO stimulation [134].

RSPO1-liposomes may also be e an effective approach to target LGR5 CSCs. RSPO1 decorated with liposomes have been constructed to interact with LGR5 via WNT/β-catenin pathway. In an *in vivo* CRC study, doxorubicin-bound RSPO1-liposomes were more effective at lower concentrations than unbound doxorubicin [135]. RSPO1-liposomes were able to target more CSC cells and deliver more drugs precisely and efficiently in mice models [135]. Even at low concentrations, doxorubicin bound with RSPO1-liposomes leading to massive tumor tissue necrosis and inhibition of LGR5+ cells growth [135]. A recent study developed a targeted therapeutic approach using the interaction between RSPO1 and LGR5 [140]. This study focused on RSPO1 receptor-binding domain (Fu1-Fu2) that deliver cytotoxic agents or immune-modulating therapies directly to LGR5-expressing ovarian cancer cells and was very effective in a LGR5-rich ovarian cancer xenograft model [140].

Chimeric antigen receptor T (CAR-T) cell immunotherapy targeting LGR5 is an emerging field of cancer treatment. We recently demonstrated that LGR5-CAR-T cells exhibited anti-tumor activities against ovarian cancer cell lines, primary HGSOC cells [133], and CRC cell lines *in vitro* and *in vivo *[141]. In CRC, LGR5-targeting CAR-T cell therapy for human metastatic CRC had minimal off-target effects [141] and are being evaluated in human clinical trials (Clinical Trials ID. NCT05759728). CAR-T cells targeting LGR5 may provide long-term protection against LGR5+ tumors as they may cooperate with the TME to decrease immune escape.

Most LGR5 inhibitors or drugs targeting LGR5 regulate the LGR5/WNT/β-catenin signaling pathway. Although most strategies showed promising results, research in this field is very limited. CAR-T cell immunotherapy targeting LGR5 has moved to clinical trials for CRC, which has the potential to be developed into novel cancer treatment for patients.

## Discussion and conclusion

LGR5 promotes WNT/β-catenin signaling pathway in brain, breast, cervical, colorectal, hepatic, gastric, and ovarian cancer. LGR5 has also been investigated as a CSC marker and associated with other CSC markers, such as CD33 and CD44 [95]. LGR5 is associated with enhanced proliferation in many cancers including brain, breast, bone, cervical, colorectal, gastric, hepatic, and lung cancer. LGR5 promoted tumorigenesis in breast, colorectal, and gastric cancer. Studies have also shown that LGR5 promoted migration, invasion and/or metastasis of breast, colorectal, oesophagus, gastric, hepatic, lung, and thyroid cancer. Increased LGR5 expression is associated with enhanced cancer migration, invasion, metastasis, and angiogenesis. In contrast, some studies have also shown that LGR5 plays an anti-tumorigenic role in brain, bone, colorectal, gastric cancer, and ovarian cancer. Disparate findings observed between LGR5 and cancer progression may be due to variations in experimental methods used and studies among different cancer types, which requires further evaluation. LGR5 + cells can interact with TGFβ which has dual roles in cancer progression, and further research is required to increase our understanding of the role of LGR5 in balancing WNT/β-catenin signaling pathways.

This review focused on the functional roles of LGR5 in cancers. LGR5 is involved in embryo repair and tissue development via WNT/β-catenin pathway which supports cell proliferation, differentiation, and tissue regeneration. LGR5’s regulatory role highlights its importance not only in embryogenesis but also in cancerous tissue regeneration. Although the majority of LGR5 studies in cancers showed that high LGR5 expression was related with tumor growth and metastasis, some studies found the opposite findings and high *LGR5* expression was associated with reduced tumor proliferation and migration, and increased patient survival. The inconsistency between protein and mRNA results may be due to individual genetic differences among patients which contributes to varied responses to LGR5 expression. Genetic polymorphisms and mutations in the *LGR5* gene among individuals can alter the function of the LGR5 gene or its expression levels, leading to diverse biological outcomes [19, 142]. Moreover, mutations in components (RSPO1 and RNF43) of the WNT signaling pathway, which is closely associated with LGR5, may also impact tumor behaviour and treatment response [143]. For instance, mutations in the APC gene or β-catenin, both of which are key players in WNT signaling, can modify the effect of LGR5 expression on tumor growth. [142, 144]. These alterations might either exacerbate the oncogenic effects of LGR5 or mitigate them, depending on the nature of the mutation and its impact on the pathways. Tumors with hyperactive RSPO1 signaling may show resistance to therapies targeting other parts of the WNT pathway [145]. RNF43-mutant cancers may resist therapies targeting downstream WNT components, as upstream overactivation remains unchecked [146]. Studies on genetic mutations and polymorphisms in the LGR5 gene are limited and require further investigation.

Standardizing the measurement of LGR5 mRNA and protein expression is crucial for consistent and reliable results across different experiments or studies. Currently, there is no approved standard method to measure or define high and low LGR5 expression. Future studies need to define a Standard Operating Procedures for both mRNA and protein expression. Large patient cohorts will need to be investigated, using normalization strategies to account for potential variations.

LGR5 has shown to be related with chemoresistance in many cancers. LGR5 enhances chemo-resistance through the WNT/β-catenin signaling pathway in cervical cancer. High expression of LGR5 in ovarian cancer cells was associated with carboplatin, cisplatin, and taxol resistance. In addition, high LGR5 expression was associated with recurrence or relapsed in breast, cervical, and colon cancers. However, two studies found high *LGR5* mRNA expression was negatively associated with platinum-sensitivity [61, 62]. The contrary findings may be due to different cancer types, the multifactorial nature of drug resistance and the complex ovarian cancer heterogeneity. Further studies are required to investigate the roles of LGR5 in chemo-resistance.

Several LGR5 inhibitors have been identified in the literature, including 4-AAQB which negatively regulated the stem cell maintenance via the LGR5/WNT/β-catenin signaling pathway. Other LGR5 inhibitors including curcumin and trichosanthin also repress the WNT/β‑catenin signaling pathway. VJ is a highly potent anticancer drug that suppresses tumor growth and metastasis and has also been shown to reduce LGR5 expression *in vivo.* Therapeutics that target LGR5-positive cells, such as ADCs, have shown promising results in preclinical studies. CAR-T cell immunotherapy targeting LGR5 has shown anti-tumor effects *in vitro* and *in vivo* in colon cancer and is now in clinical trial for CRC [141]. LGR5-targeted therapies can selectively kill LGR5-expressing CSCs without harming normal cells that lack high LGR5 expression. This specificity may minimize off-target effects and increase treatment efficacy in cancers heavily reliant on LGR5-driven signaling for growth and survival. Although several drugs targeting LGR5 have shown anti-tumor effects there are many challenges when targeting LGR5 in cancer treatments. It is difficult to produce a stable and effective LGR5-targeting cancer treatment as LGR5 is involved in both WNT and TGFβ pathways which has a dual role in modulating tumor progression. CSC populations expressing LGR5 can exhibit plasticity, potentially shifting expression in response to therapies and leading to therapeutic resistance and relapse. Safety is another obstacle because LGR5 is also found in normal adult stem cells, such as the intestinal crypts. Targeting LGR5 might unintentionally damage these cells, raising concerns about potential gastrointestinal side effects. Thus, while LGR5 presents a promising target for innovative cancer therapies, advancing its application requires further research to refine targeting strategies and address challenges associated with tumor heterogeneity and delivery.

In conclusion, LGR5 has both tumor-promoting roles and tumor-inhibitory roles in many cancer types. LGR5 is involved in the WNT signaling pathway and associated with chemo-resistance/recurrence in cancer. Drugs targeting and inhibiting LGR5 can suppress tumor growth by regulating LGR5/WNT/β-catenin signaling pathway. Both LGR5 + and LGR5- CSC cells may need to be targeted at the same time due to the LGR5-involved in the TGFβ pathway. CAR-T cell therapy targeting LGR5 is a promising therapy for chemoresistant cancer patients, which requires further investigation.

## Data Availability

No datasets were generated or analysed during the current study.
